# The Role of Wisdom in the Cross-Cultural Adaptation of Chinese Visiting Scholars to Canada: A Mediation Model

**DOI:** 10.3389/fpsyg.2022.779297

**Published:** 2022-03-25

**Authors:** Dan Bao, Liqing Zhou, Michel Ferrari, Zhe Feng, Yahua Cheng

**Affiliations:** ^1^School of Humanities and Social Sciences, Hubei University of Medicine, Shiyan, China; ^2^School of Education, Hangzhou Normal University, Hangzhou, China; ^3^Department of Applied Psychology and Human Development, University of Toronto, Toronto, ON, Canada; ^4^Department of Psychology, Ningbo University, Ningbo, China

**Keywords:** wisdom, coping, adaptation, life satisfaction (SWLS), Chinese visiting scholars

## Abstract

This study examines the role of wisdom in the cross-cultural adaptation of Chinese visiting scholars in Canada, as mediated by different coping styles. Path analysis was used to for hypotheses testing. The findings suggest that (1) wisdom measured by 3D-WS and Adult Self-Transcendence Inventory (ASTI), independently had direct correlation with social and psychological adaptation, and positively associated with engaged coping (active coping and proactive–reflective coping); (2) the independent effects of 3D-WS and ASTI on social adaptation, psychological adaptation, and life satisfaction were mediated by proactive–reflective coping; (3) wisdom, when measured by 3D-WS, promoted positive psychological adaptation through decreasing passive coping. This study shows that wisdom is a critical factor affecting cross-cultural adaptation, and the use of proactive–reflective coping is a wise way of handling future life challenges.

## Introduction

### The Science of Wisdom

Wisdom is considered an ideal endpoint of human development around the world. According to Erikson (1950), wisdom arises out of successful resolution and integration of various psychosocial conflicts that allow for better psychosocial adaptation. As a result, regardless of objective circumstances, wisdom is often related to a higher level of life satisfaction (Ardelt, 1997; Tornstam, 1997). Wise individuals are those who can integrate feelings, thoughts, and behaviors across intrapersonal, interpersonal, and transpersonal domains (Orwoll and Perlmutter, 1990; Orwoll and Achenbaum, 1993; Sternberg et al., 2019) and brilliantly apply their knowledge and judgment in real-life contexts. In general, wisdom is manifested in one’s way of handling daily events, managing one’s own life, making critical life decisions, and taking wise actions that would exert a significant and positive effect on human life.

In spite of different specific definitions of wisdom, most researchers—East and West—agree on the multidimensional nature of wisdom as an integration of knowledge, emotion, and virtue (Clayton and Birren, 1980; Randall and Kenyon, 2001; Baltes and Smith, 2008; Wang and Zheng, 2014; Ardelt and Ferrari, 2019). Additionally, by distinguishing first and third-person perspectives (Searle, 1992), psychological wisdom research can be differentiated into general and personal wisdom (Staudinger, 1999; Staudinger and Glück, 2011; Staudinger and Leipold, 2019). Personal wisdom relates to insight into one’s own self and life through personal experiences and insights, whereas general wisdom concerns the more detached insight into the lives of others and human life in general (Glück et al., 2013; Staudinger, 2013).

Since these different types of wisdom studies have different purposes, the measurement of personal wisdom should be distinguished from that of general wisdom. Thus, in this study, we choose to measure Chinese visiting scholars’ personal wisdom using the three-dimensional Wisdom Scale (3D-WS) of Ardelt (2003)—the combination of cognitive, reflective, and affective characteristics, and the Adult Self-Transcendence Inventory (ASTI) that focuses on self-transcendent wisdom (Orwoll and Perlmutter, 1990; Levenson et al., 2005). The former refers to more interpersonal domain, and the latter refers to a transpersonal and intrapersonal domain.

Based on lay and expert wisdom theories (Clayton and Birren, 1980; see also Weststrate et al., 2019), Ardelt proposed a self-report wisdom measure and identified three personal dispositions jointly required for wisdom (Ardelt, 1997, 2003; Ardelt and Ferrari, 2014). The cognitive dimension measures the individual’s deep understanding for truth and knowledge or experience of life. The reflective dimension refers to the individual’s willingness and ability to perceive reality and examine oneself from different angles, which implies self-examination and self-insight. The affective dimension reflects the individual’s sympathy and compassion for others (Ardelt, 2003; Glück, 2017).

In addition, this scale nicely captures both Western and Eastern implicit and explicit wisdom theories. Based on the work by Clayton and Birren (1980), the lay conceptions of wisdom described as cognitive (knowledgeable, experienced, intelligent, pragmatic, and observant), reflective (introspective and intuitive), and affective (understanding, empathetic, peaceful, and gentle), Holliday and Chandler (1986) also named the factors “exceptional understanding” as a mixture of reflective, “general competencies” and “judgment and communication skills” as mixture of cognitive, “interpersonal skills” as a mixture of affective. Similarly, from Sternberg’s theory (Sternberg, 1990a), “reasoning ability,” “sagacity,” “learning from ideas and environment,” “judgment,” “expeditious use of information,” and “perspicacity,” all of the dimensions contain cognitive, reflective, and affective wisdom attributes. These core components outlined above are all critical features of lay view (Glück and Bluck, 2011; Staudinger, 2013).

The scale of 3D-WS is also with explicit theories that are based on the Western and Eastern wisdom traditions (Takahashi and Bordia, 2000). The wisdom traditions of the West emphasize the cognitive dimension of wisdom (i.e., knowledge and analytical ability), Baltes and colleagues (Baltes and Smith, 1990; Baltes and Staudinger, 2000) defined wisdom as “an expert knowledge system in the domain, fundamental life pragmatics.” In the Eastern wisdom traditions, wisdom is characterized by flexibility, honesty, sensitivity, modesty, compassion, morality, and a balanced state of mind that is able to perceive and accept the reality of the present moment (Clayton and Birren, 1980; Hart, 1987; Nakasone, 1994; Levitt, 1999; Yang, 2014a; Wang and Zheng, 2014). Likewise, the explicit theory of Yang (2001, 2014b) and model of wisdom includes four factors, namely, (1) competencies and knowledge, (2) modesty and unobtrusiveness, (3) openness and profundity, and (4) benevolence and compassion. Furthermore, wisdom is considered a real-life process with three core elements: cognitive integration, embodied action, and positive effects for self and others. Wang and Zheng (2015) proposed an indigenous Chinese wisdom theory, where wisdom is defined as the integration of virtue and competence. Here, personal wisdom is manifested in daily life through processes that often involve a high level of intellectual competence (e.g., dialectical thinking, reflective thinking, innovative thinking, and critical thinking) and a high level of morality (e.g., moderation, honesty, responsibility, fairness, and compassion; Chen and Wang, 2014).

In general, all dimensions of 3D-WS can relate to the mainstream explicit wisdom theories from West and East.

Based on an analysis of the common elements of wisdom definitions provided in different cultures, Curnow (1999) first put forward self-transcendence as the essence of wisdom and identified four major features of wisdom: self-knowledge, integration, self-transcendence, and detachment, considered stages in the development of wisdom (Levenson et al., 2005). In terms of self-transcendence, the core feature is to transcend human biases, subjectivity, and self-centeredness. In turn, this can result in critical self-reflective knowledge, in line with the reflective dimension of the 3D-WS. In a recent study, Glück et al. (2013) found that the ASTI overlapped with a broad range of subscales of other wisdom measures including the 3D-WS (Ardelt, 2003), which assess several wisdom dimensions that have relationships with the sub-dimensions of the ASTI. However, these correlations are more strongly supported with North American and European samples, and not replicated in China or elsewhere.

Glück et al. (2013) compared four well-established wisdom measures: the SAWS (Webster, 2003), the 3D-WS (Ardelt, 2003), the ASTI (Levenson et al., 2005), and the Berlin wisdom paradigm (Baltes and Staudinger, 2000). Based on analysis of the content of researchers’ definitions and operationalizations of wisdom, the ASTI and the 3D-WS predominantly pertain to personal wisdom. Besides, the cognitive dimension of the 3D-WS also taps general wisdom, and an affective dimension of the 3D-WS relates to another important aspect of wisdom which refers to empathy-based concerning, namely, other-related wisdom. The ASTI presents its core aspect of wisdom as self-transcendence, which seems narrower than two other self-report scales, but the findings from this study support the idea that it taps a wide range of meanings of wisdom and self-transcendence is central to wisdom across all measures. Thus, this study adopts the 3D-WS and the ASTI which, combined, largely capture universal features of wisdom as mentioned above (Glück et al., 2013). Both measures of personal wisdom have been widely used in the international sample of different ages with relatively good overall reliability and validity (Ardelt, 2003; Levenson et al., 2005; Webster, 2007; Taylor et al., 2011; Thomas et al., 2017). They represent the two ways people may respond optimally to being a sojourner.

However, due to the major differences between Western and Eastern understandings of wisdom (Takahashi and Bordia, 2000; Birren and Svensson, 2005; Takahashi and Overton, 2005), Historically, Western wisdom is said to value knowledge and cognitive complexity through sophisticated reasoning and judgment, while Eastern wisdom has a more nuanced discussion of virtuous action (Cottingham, 1996; Magee, 1998; Takahashi and Bordia, 2000; Takahashi and Overton, 2005), so it is worth considering whether the definitions of wisdom and their components are universal in different cultures and religious traditions. Although the differences between Eastern and Western wisdom conceptions and assessments have been compared in some studies (e.g., Takahashi and Bordia, 2000; Yang, 2001; Takahashi and Overton, 2005; Kim and Knight, 2015), there is limited empirical evidence from Eastern countries.

### Wisdom, Coping, and Acculturation

Acculturation refers to one’s psychosocial adaptation to a culture different from one’s native culture (Burnam et al., 1987). It is closely associated with sociocultural adaptation (Ward and Kennedy, 2001), acculturative stress (Kuo and Roysircar, 2004; Torres and Rollock, 2004) among others (Yoon et al., 2012). Many of the conceptual paradigms of acculturation proposed by several researchers share the similarity in that they include both internal and external domains (Kwan and Sodowsky, 1997). The internal domain usually refers to the psychological aspects of an individual’s orientation toward their larger cultural group and society, whereas the external domain usually refers to the behavioral aspects of an individual’s orientation toward their ethnic cultural group (Chia and Costigan, 2006). According to Berry (2006), there are two distinct perspectives of migrants’ cultural adaptation, namely, the cultural learning model (behavioral adaption, or doing well) and the “stress, coping, and adaptation” model (experience, or feeling well).

Cultural learning emphasizes teaching and training migrants in culture-specific skills in order to acculturate them in the new cultural environment (Ward, 2001). It is related to multiple factors, including the length of new culture residence, foreign language proficiency, cultural knowledge, and cultural distance. In contrast, psychological adaptation to stress focuses on immigrants’ emotional wellbeing and life satisfaction in the host culture, including their ability to cope with conflict and stress (Berry, 1997, 2006; Ward, 2001). Some studies show that migrants with different acculturation experiences react differently to stresses and adopt different coping strategies (Zheng and Berry, 1991; Yoshihama, 2002; Noh and Kaspar, 2003).

Importantly, Staudinger (2001) defined coping as social-cognitive processes of life reflection (i.e., life planning, life management, and life review) which were assumed to be essential for the development of wisdom-related knowledge and judgment. Reflection has been recognized as a core element of most definitions of personal wisdom (e.g., 3D-WS and ASTI) and a critical characteristic of wise individuals (Weststrate et al., 2019), emphasizing the motivation to think deeply and take different perspectives on experiences. Self-transcendent wisdom is deep transcendental insight into one’s self and the nature of reality, which primarily focuses on specific and basic existential issues like identity, reality, life purpose, meaning, and priorities (Staudinger, 2013). In addition, Linden et al. (2011) argued that wisdom could be understood as a psychological competency in coping with difficult, ambiguous, or unsolvable life problems, which helps people to tolerate or cope with complex and uncertain life situation in the future (Linden, 2014).

Recently, attention has been shifted to future-oriented proactive coping, that is, how one faces life instead of reacting to it after-the-fact. It includes one’s efforts to build up general resources to buffer future changes, address challenges, and facilitate personal growth (Safdar et al., 2009a). Safdar et al. (2003, 2009a,b, 2012, 2013) found that engaged coping strategies (i.e., meaning-focused and proactive coping) for acculturation-specific and generally stressful life-planning challenges, predict mental and physical health outcomes.

Foresight is also an increasingly acknowledged element in many lay theories and models of wisdom (Holliday and Chandler, 1986; Ji et al., 2001; Asadi, 2015; Weststrate et al., 2019; Ardelt et al., 2020). Some studies have found that wise reasoning about future conflicts better resolves conflicts (Huynh et al., 2016; Peetz and Grossmann, 2020), and training wise reasoning skills results in more balanced predictions about future emotional reactions (Grossmann et al., 2020; Peetz and Grossmann, 2020).

From the brief literature review above, we can see that wisdom is “a special coping strategy in which the individual brings together whatever intellectual and affective strengths are available to them to resolve a unique problem” (Bluck and Glück, 2004, p. 548). Some key characteristics of wisdom in response to real-life challenges involve self-transcendent meta-level responses (epistemic/intellectual humility, multi-angle considerations, appreciation of a given issue’s broader context) with moral grounding (Grossmann et al., 2020). Furthermore, wisdom is often considered to be the result of reflecting on life experiences, particularly challenging ones (Aldwin, 2007; Weststrate and Gluck, 2017; Munroe et al., 2021), manifested through expert life management and decision-making when dealing with stressful situations, social relationships, life tasks and goals, and life’s uncertainties (Baltes and Smith, 1990).

There have been some empirical studies of wisdom in the acculturation of ethnic minorities. Typically, ethnic minority research explores power disparities and their implications for equal access to social resources, investigating individuals’ psychological experiences through the understanding of the obstacles to be overcome, as well as the strengths on which they depend to do so (Coll et al., 1996). For example, Le (2008) found that European Americans exhibited a higher level of transcendent and practical wisdom than Vietnamese Americans. However, the wisdom experiences of Vietnamese refugees living in the United States might reflect the traumatic life experience of these immigrant participants as well as any difference between the Vietnamese culture and the US culture (Le, 2008, 2010). More universally, wise coping may help migrants better resolve psychological difficulties during acculturation process and lead to eventual adaptation in the host culture (Berry, 2006). People have the potential to use a wide range of coping strategies to achieve various outcomes (adaptations). Examining international students in Singapore, studies found that secondary coping mechanisms (e.g., acceptance and positive reinterpretation) predicted lower levels of perceived stress and subsequently reduced depressive symptoms (Ward et al., 1998, as cited in Ward and Kennedy, 2001). Similarly, Lin and Betz (2009) discovered a negative correlation between unconditional positive regard (i.e., self-compassion) and acculturative stress among Chinese international students.

To sum up, wise ways of dealing with stressful situations are theorized to be critical determinants of acculturation. Wise individuals acknowledge that values are relative, there is rarely absolute certainty, and contradiction is inherent in life. However, previous research is limited to immigrants who, with different acculturation needs, are qualitatively different from visiting scholars which are the interest of the present study.

### International Cultural Exchange (Long-Term Visitors)

While many countries have programs that enable international professional exchanges, one of the largest groups is from mainland China. As the global knowledge economy is increasingly characterized by competition and internationalization, China has taken the lead in sending the largest proportions of visiting scholars abroad [Institute of International Education (IIE), 2019].^1^ Chinese visiting scholars are people from an academic institution, who visit a host university in the international culture to perform research on a topic the visitor is valued for. The groups are composed of China’s post-secondary institution researchers, faculty members, post-doctoral fellows, and backbone teachers. In recent years, China has implemented national policies to encourage the development of academic professionalism of its scholars and promote the cooperation between colleges in China and other countries. Post-secondary institutions are required to encourage global exchanges and visits which are often of great importance for faculty members who are interested in career advancement. Thus, the number of China’s visiting scholars who visit foreign countries to obtain global experiences has been raising every year, from 2,044 in the first year of CSC project that had been launched (Xue et al., 2015) to 12,900 participants by 2018 (China Scholarship Council, 2018). A visiting scholar can stay for a few months to a year in the host country, and stays may be extended as required by study programs. However, a visiting scholar and his/her family members are just temporarily exposed to the host culture since they will come back to China after the visiting period. At the same time, the visiting scholars have to face cultural challenges in behaviors and attitudes as long as the host culture interacts with their culture of origin. Triandis found that Westerners advocate individualism, while Easterners are on the collectivism end (Triandis, 1989; Singelis et al., 1995; Triandis and Gelfand, 1998). Nisbett and Peng conducted in-depth research on the differences between Chinese and Western thinking pattern, it is indicated that Western culture shows an analytic thinking orientation, while Chinese people show a holistic thinking orientation, etc. (Peng et al., 1997; Nisbett et al., 2001; Nisbett and Masuda, 2003; Masuda and Nisbett, 2006), besides Westerners emphasize more on independent self while Easterners tend to be interdependent self. Those deep cultural differences may have a significant impact on the social and psychological adaptation of Chinese visiting scholars in Canada.

Moreover, they may have different and similar acculturation experiences that are comparable to those of Chinese immigrants or international students, for instance, limited cross-cultural competence, culture shock, academic adaptation, and language barriers (Mori, 2000; Kim and Abreu, 2001; Lee et al., 2004; Russell et al., 2010). However, little has been done to explore the adaptation experiences of China’s visiting scholars. A survey of international visiting scholars’ experiences, especially for their cross-cultural challenging experiences, have the possibility of helping them to cope with or tolerate the complex and ambiguous situations in foreign countries, which is informative not only to China, but policy-makers, educators, professionals, and students of other countries. This study intends to offer practical suggestions and supports for visiting scholars in terms of pre-departure preparations, goal settings, and plans to maximize their exchange experience.

### Current Study

This study examines the effects of coping styles (proactive–reflective, active, passive) as mediators between wisdom and adaptation among Chinese visiting scholars. Considering the strong theoretical links between wisdom and coping strategies, and the important role of wisdom in adaptation and wellbeing, we propose the following hypotheses:

1. Facing challenging and stressful life experiences during the exchange period, wisdom, as measured by the 3DWS and the ASTI, directly predicts adaptation and life satisfaction.
2. People with wisdom as measured by the 3DWS or the ASTI will differentially endorse different coping strategies (proactive–reflective, active, passive), and in turn exhibit different levels of social and psychological adaptation. Specifically, people who score higher on wisdom measures may show more engaged coping (proactive–reflective and active coping strategies) that help them acculturate more easily in Canada.
3. People who score higher on wisdom measures will have less psychological distress.

## Materials and Methods

### Participants and Procedure

Two hundred and one Chinese visiting scholars temporarily residing in Canada participated in the study. Hundred eighty-one usable responses were included for the final analyses, 20 were excluded because of their visiting period was not included in pandemic period or incomplete data for several key information (e.g., age and visiting period). Fifty-three (53%) of the participants were men, with age ranging from 22 to 59 years (*M* = 35.11, *SD* = 6.11). Among them, 124 (68.5%) were married, 49 (27.1%) were unmarried, 7 (3.9%) were divorced, and 1(0.5%) was widowed. The mean length of stay in Canada was 11.93 months (*SD* = 5.08). The length of residence ranging from 1 to 6 months was 3.9%, 7 to 12 months was 78.4%, and above 12 months was 17.7%.

The questionnaire was piloted with four Chinese visiting scholars through the online survey platform SoJump. During the formal data collection, through advertisements posted on WeChat groups, 201 participants consented and completed the survey through SoJump, and were paid 10RMB through WeChat’s red packet function.

### Measures

The survey collected participants’ demographic data (gender, age, marital status, months of stay in Canada, previous exchange experiences, visiting purpose, English proficiency, and the perceived impact of COVID-19), responses to two wisdom measures (3D-WS and ASTI), coping styles, adaptation, and life satisfaction. All the measures were used in Chinese version for survey.

#### 3D-WS

The adapted Chinese version of the 3D-WS was used to measure participants’ wisdom (Fu, 2019). The 39-item questionnaire consists of 14 cognitive, 12 reflective, and 13 affective items that have been applied to many culturally diverse samples (Benedikovicova and Ardelt, 2008; Bergsma and Ardelt, 2012; Bang and Montgomery, 2013; Glück et al., 2013; Bang and Zhou, 2014; Sahrani et al., 2014). Items were measured on a 5-point Likert scale, with higher scores indicating a higher level of wisdom development. In this study, the Cronbach alpha coefficient for 3D-WS was 0.94.

#### Adult Self-Transcendence Inventory

The ASTI (Koller et al., 2017) consists of 24 Likert scale items ranging from 1 (disagree strongly) to 4 (agree strongly), which conceptualizes wisdom as self-transcendence and to reflects a “decreasing reliance on externals for definition of the self, increasing interiority and spirituality, and a greater sense of connectedness with past and future generations” (Levenson et al., 2005). In this study, the Cronbach alpha coefficient for ASTI was 0.91.

#### Active and Passive Coping

The 20-item Simplified Coping Style Questionnaire (SCSQ) measures the tendency to which individuals respond to stress (Xie, 1998), including 12 items on active coping and 8 items on passive coping. All items are scored on a 4-point Likert scale (1 = do not do this at all, 4 = do this a lot) with higher scores indicating greater active/passive coping manners. In this study, the Cronbach alpha coefficients for active and passive coping were 0.87 and 0.81, respectively.

Proactive–reflective coping. The version of Proactive Coping Inventory (Greenglass et al., 1999) is a multidimensional coping inventory. The 14-item proactive coping and 11-item reflective coping subscales of the PCI were used to assess proactive and reflective coping. Participants were asked to indicate on a 4-point scale (1 = not at all true, 4 = completely true), the proactive coping scale combines autonomous goal setting with self-regulatory goal attainment cognitions and behavior, and the reflective coping scale describes simulation and contemplation about a variety of possible behavioral alternatives by comparing their imagined effectiveness and generating hypothetical plans of action. The correlation of the proactive coping scale average score to the reflective coping scale average score was 0.71.

In this study, we used Chinese version of the proactive coping scale and the reflective coping scale (Cheng and Greenglass, 2006; Wu et al., 2008), a single score was derived from these two subscales and was named proactive–reflective coping. The Cronbach alpha coefficient for our sample was 0.92.

#### Social Adaptation

Measurements of social adaptation consist of a 13-item Sociocultural Adaptation Scale (SAS, Ward and Kennedy, 1999) and a 9-item self-made work adaptation scale. The original SAS was made up of 41 items covering a variety of social situations and requires respondents to indicate the degree of difficulty they encounter in those social situations using a 5-point scale. An evolving program of cross-sectional research has convincingly demonstrated that the SAS is a reliable and valid measurement of cultural competence or behavioral adaptability in cross-cultural sojourners (Gao, 2014). In our study, some modifications have been made to the questionnaire and fewer items were included, as fitting the range of experiences of visiting scholars. Altogether five dimensions (interpersonal relationship, public situations, academic situations, academic achievement, social support) were included based on the original items. The Cronbach alpha coefficient for social adaptation was 0.75 in our sample.

#### Psychological Adaptation

The Zung (1965) Self-Rating Depression Scale (ZSDS) consists of 20 items that measure affective, cognitive, and physiological components of depression. Participants respond on a 5-point scale (1 = disagree strongly, 5 = agree strongly) and higher scores are associated with greater depression. It has been used extensively in cross-cultural research (Zung, 1969) and has consistently proven reliable and valid in multinational sojourner studies (e.g., Ward and Kennedy, 1999; Zhu, 2011; Gao, 2014). In this study, the Cronbach’s alpha coefficient for psychological adaptation was 0.86.

#### Life Satisfaction

The Satisfaction with Life Scale (SWLS) was designed to assess the respondent’s overall judgment of their life in order to measure the concept of life satisfaction (Diener et al., 1985). The 5-item SWLS are global rather than specific in nature, allowing respondents to weight domains of their lives in terms of their own values (Frisch et al., 1992). In this study, the Cronbach alpha coefficient for SWLS was 0.90.

### Common Method Bias Test

All data in this study were collected by the self-report method, which may lead to common method bias. We first controlled common method bias from measurement procedures, such as reverse-coded items and anonymous questionnaire filling. Then, Harman’s single-factor test was carried out before formal data analyses ((Podsakoff et al., 2003). The results showed that the total number of factors with eigenvalues greater than 1 was 37, and the variance of the first factor was 15.79% which was less than the critical value of 40%. Therefore, there was no obvious common method bias in the data of this study.

### Data Analyses

Testing the hypotheses, structural equation modeling (SEM) was employed to investigate the relationship between wisdom and adaptation as mediated by coping styles using Mplus7.11, which provides the maximum likelihood estimation with robust standard errors (MLR). The model chi-square (*χ*^2^), *χ*^2^ to df ratio (*χ*^2^/df), the comparative fit index (CFI), Tucker–Lewis index (TLI), the standardized root mean square residual (SRMR), and the root mean square error of approximation (RMSEA) were used to evaluate the model fit. The combination of model chi-square values accompanying *p* > 0.05, *χ*^2^ to df ratio < 2, CFI and TLI values>0.95, RMSEA and SRMR values<0.05 indicated a good model fit. Other researchers have discussed that CFI values between 0.90 and 0.95 are an acceptable fit (Kline, 2011).

## Results

### Correlation Analysis

Tables 1 and 2 present the descriptive statistics and correlations. Pearson’s correlation analyses (Table 2) indicated that 3D-WS and ASTI were significantly and positively correlated with social adaptation and psychological adaptation, but only ASTI was significantly correlated with SWLS. Active coping and proactive–reflective coping were both significantly and positively correlated with social adaptation, psychological adaptation, and SWLS; passive coping was negatively correlated with psychological adaptation but positively correlated with SWLS. 3DWS was positively correlated with active coping and proactive–reflective coping, but negatively correlated with passive coping. ASTI was positively correlated with active coping, passive coping, and proactive–reflective coping. Proactive–reflective coping was highly correlated with active coping. The correlation analysis provided evidence to support the test of mediation model.

**Table 1:** Minimum, maximum, means, SDs, skewness, kurtosis, reliability.

Variables	Minimum	Maximum	M	SD	Skewness	Kurtosis	Reliability
1. Age	22.00	59.00	35.11	6.11	0.80	1.11	
2. Visiting period	1.00	49.00	11.93	5.08	2.91	16.43	
3. English competence	1.00	3.00	2.59	0.63	−1.26	0.46	
4. COVID-19 effect	1.00	6.40	3.02	0.83	−0.29	1.09	0.89
5. 3DWS	2.03	4.45	3.19	0.47	0.04	0.06	0.94
6. ASTI	2.17	5.00	3.61	0.51	−0.11	0.15	0.91
7. Active coping	2.00	4.00	3.10	0.42	0.01	0.00	0.87
8. Passive coping	1.25	4.00	2.56	0.55	0.40	−0.12	0.81
9. Proactive–reflective coping	1.64	3.92	3.02	0.37	0.14	0.85	0.92
10. Social adaptation	2.68	4.41	3.41	0.36	0.41	−0.01	0.75
11. Psychological adaptation	2.35	5.00	3.62	0.53	0.21	−0.71	0.86
12. SWLS	1.00	5.00	3.40	0.71	−0.32	0.20	0.90

**Table 2:** The correlation matrix among variables.

Variables	1	2	3	4	5	6	7	8	9	10	11	12	13
1. Gender	1												
2. Age	0.09	1											
3. Visiting period	−0.08	−0.14	1										
4. English competence	−0.10	−0.09	−0.06	1									
5. COVID-19 effect	−0.09	−0.06	0.15^*^	0.02	1								
6. 3DWS	−0.09	0.17^*^	0.09	0.03	0.24^**^	1							
7. ASTI	−0.03	0.06	0.10	−0.08	−0.13	−0.07	1						
8. Active coping	0.11	0.09	0.05	−0.18^*^	−0.10	0.20^**^	0.41^**^	1					
9. Passive coping	0.09	−0.09	0.03	−0.03	−0.16^*^	−0.59^**^	0.17^*^	0.22^**^	1				
10. Proactive–reflective coping	−0.07	−0.06	0.13	−0.14	−0.07	0.30^**^	0.39^**^	0.63^**^	0.02	1			
11. Social adaptation	−0.07	0.03	0.17^*^	−0.19^*^	0.07	0.37^**^	0.41^**^	0.40^**^	−0.11	0.58^**^	1		
12. Psychological adaptation	−0.03	0.22^**^	0.01	−0.08	0.14	0.60^**^	0.22^**^	0.35^**^	−0.44^**^	0.46^**^	0.56^**^	1	
13. SWLS	0.09	−0.07	−0.00	−0.09	−0.06	−0.05	0.32^**^	0.35^**^	0.25^**^	0.36^**^	0.37^**^	0.12	1

*N = 181*.

* *p < 0.05*;

** *p < 0.01, two-tailed*.

### Path Analysis

**Figure 1:**
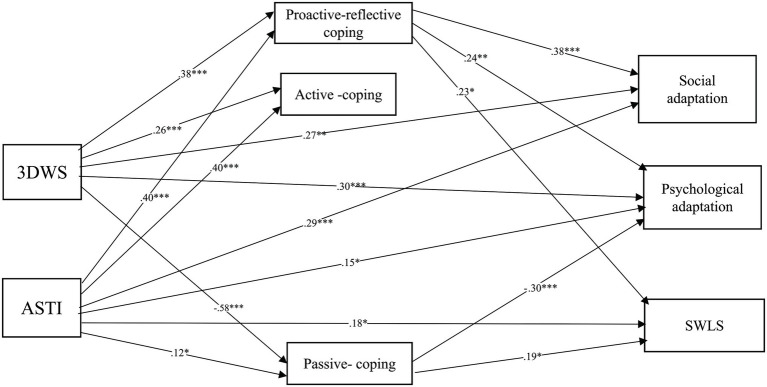
Standardized parameter estimates of the effects of wisdom [3DWS and Adult Self-Transcendence Inventory (ASTI)] on adaptation and SWLS mediated by coping styles. *N* = 181, Model fit: *χ*^2^ = 14.73, df = 11, *χ*^2^/df = 1.34, CFI = 0.99, TLI = 0.97, root mean square error of approximation (RMSEA) = 0.04, root mean square residual (SRMR) = 0.04, ^*^p < 0.05, ^**^p < 0.01, ^***^p < 0.001. SWLS = life satisfaction.

We applied path analysis to test the proposed mediation model as depicted in Figure 1, with estimates of the standardized path coefficients.

The mediation model showed a good fit, *χ^2^*(11) =14.73, *p* = 0.20 (*χ^2^*/*df* = 1.34), CFI =0.99, TLI =0.97, RMSEA =0.04 (90% CI = 0.00–0.09), SRMR =0.04. The effects of 3DWS and ASTI on social adaptation, psychological adaptation, and SWLS mediated by proactive–reflective coping were significant. 3DWS and ASTI negatively affected psychological adaptation but positively affected SWLS through passive coping. The paths from 3DWS and ASTI to social adaptation, psychological adaptation, and SWLS, as mediated by active coping were not significant.

We then examined the direct path coefficient from 3DWS and ASTI to social adaptation, psychological adaptation, and SWLS, without the mediators, after controlling for gender, age, visiting period, English proficiency, and the perceived impact of COVID-19. Results showed that the direct path coefficients from ASTI to social adaptation, psychological adaptation, and SWLS were statistically significant (*β* = 0.29, *p* < 0.001, *β* = 0.15, *p* < 0.05, *β* = 0.18, *p* < 0.05), and the direct path coefficients from 3DWS to social adaptation and psychological adaptation were statistically significant (*β* = 0.27, *p* < 0.01, *β* = 0.30, *p* < 0.001).

To evaluate the significance of indirect effects, bootstrapped standard errors were calculated (a bootstrap sample of 5,000 was specified) for the parameters and used to construct 95% confidence intervals (CIs). This was done because *z* statistics for indirect effects can be biased by deviations from distributional assumptions (MacKinnon, 2008). Significance of indirect effects on bootstrapping data is determined by whether zero is included within the 95% confidence interval. Confidence intervals including zero are considered non-significant. We estimated all possible indirect effects and the associated 95% bootstrap confidence intervals in the model. As displayed in Table 3, 3DWS and ASTI both exerted effects on social adaptation, psychological adaptation, and SWLS, through the indirect path *via* the mediating effect of proactive–reflective coping. Additionally, 3DWS exerted its effect on psychological adaptation through an indirect path *via* the mediating effect of passive coping.

**Table 3:** Standardized indirect effects and 95% CI for the mediation model.

Model pathways	Estimated	95% CI	
		Lower	Upper
ASTI → Proactive–reflective coping → Social adaptation	0.15^a^	0.07	0.23
3DWS → Proactive–reflective coping → Social adaptation	0.15^a^	0.06	0.23
ASTI → Proactive–reflective coping → Psychological adaptation	0.10^a^	0.03	0.17
3DWS → Proactive–reflective coping → Psychological adaptation	0.10^a^	0.02	0.16
3DWS → Passive coping → Psychological adaptation	0.17^a^	0.09	0.26
ASTI → Proactive–reflective coping → SWLS	0.09^a^	0.01	0.17
3DWS → Proactive–reflective coping → SWLS	0.09^a^	0.01	0.17

a *Bootstrap confidence intervals that exclude 0*.

## Discussion

The main objective of this study is to investigate the role of wisdom in the Chinese visiting scholars’ adaptation and life satisfaction and extend the literature by examining the mediation effects of coping styles underlying this association. Overall, the hypotheses were partially supported.

Wisdom (measured by 3DWS and ASTI) was directly correlated to social and psychological adaptation and was positively associated with engaged coping (active coping and proactive–reflective coping). The apparent positive impact of wisdom on the endorsement of engaged coping styles and adaptation may be attributed to the complex and multifaceted nature of wisdom which is a construct that involves expert life management and planning in selecting, optimizing, and compensating in the face of life challenges (Freund and Baltes, 2002a, 2002b) and manifests in how people cope with real-life problems (Glück et al., 2005; Grossmann et al., 2016). Wiser or less wise individuals act differently in how they handle difficult, uncertain, and challenging situations (Gluck and Bluck, 2013). In the face of difficulties, wiser individuals may place their lives and the challenges they encounter in a larger and meaningful context, which can facilitate positive coping. In this way, wisdom guides the coping behavior of Chinese visiting scholars. For instance, wiser individuals may apply engaged coping strategies when dealing with acculturation challenges.

Even though previous studies also suggested that high wisdom scorers were more engaged in coping with challenging events in their lives (Ardelt, 2005, 2010), in our study, only 3DWS was negatively correlated to passive coping, while ASTI showed a positive association. This may be due to the broad concept of wisdom and behavioral influence from the cultural setting. Based on the philosophical analysis of cross-cultural European and Asian wisdom, the ASTI (Levenson et al., 2005) defines wisdom as self-transcendence, that is, an inner peace independent of external disturbance and the feelings of unity with others and nature, which may have some consistency with some aspects of the Chinese Daoist philosophy. Daoism inherited identical nature-centric epistemology and methodology (Sivin, 1978). The influence of traditional Daoist attitude toward life and way of person thinking and making has been deeply imprinted in Chinese people (Feng, 2014; Tu and Guo, 2014). Wang and Wang (2021) elaborated further that in the view of the Lao-Zhuang (Daoist representatives Laozi and Zhuangzi) school of thought, Daoism values the doctrine of dao which includes the soft, weak, empty, simple, non-doing, and natural self-characteristics. Consistent with Daoist Self, one can comprehend and accept the laws of nature, live in unconstrained freedom, and take things as they come (Feng, 2014; Wang and Wang, 2020). Thus, Daoism advocates naturalness, effortlessness, and non-action, which might include some passive coping strategies, such as “I’ve been accepting the reality of the fact that it has happened” or “I’ve been thinking that time will change the status quo, the only thing to do is to wait.” Given that different cultures can shape or emphasize different dimensions of wisdom, people from different cultural backgrounds often attach importance to various aspects of wisdom (Takahashi and Bordia, 2000; Yang, 2001; Takahashi and Overton, 2005). Chinese visiting scholars’ behavior manifests the “inaction” in Chinese culture, which suggested that under the influence of Confucian and Daoist philosophy, some of the wiser individuals might choose to let things take their own course, especially during the COVID-19 pandemic.

This study also revealed that coping styles partially mediated the association between wisdom, adaptation, and life satisfaction. It is interesting to find that the path of ASTI, 3DWS → Proactive–reflective coping→ Social adaptation, Psychological adaptation, and SWLS were all significant. This indicates that wiser Chinese visiting scholars had a tendency to adopt future-oriented proactive coping strategies to prepare for potential future challenges, and in turn, it positively influences their adaptation and life satisfaction in Canada.

Coping is typically referred to as an emotional, behavioral, and cognitive efforts to manage the demands created by stress or conflict (Lazarus and Folkman, 1984). It usually occurs as a reaction to stressful events. Proactive coping is a process through which one prepares for potential future stressors and promote personal growth and wellbeing (Aspinwall, 1997; Aspinwall and Taylor, 1997). In other words, proactive–reflective coping demonstrates how an individual faces life rather than reacting to it. It is a wise way of coping and may help to prevent future threats to personal goals. Grossmann et al. explored the relationship between wisdom, future-directed reasoning, and affective forecasting. These studies found that wise reasoning about future conflicts can better help to solve conflicts (Huynh et al., 2016; Peetz and Grossmann, 2020). It is also found in the Chinese cultural sphere that those who can predict changes are considered wiser (Ji et al., 2001). The influential text *The Classic of Changes* (易经) has historically been used for diagnosing particular moments in time, including how to act wisely to avoid ill fortune. It may provide ideas to increase wisdom and make the proactive model possible (Kim et al., 2020). In addition, it was found by Mohammad and Zahra (2012) that all the proactive coping skills significantly and negatively correlated to depression. Kahana et al. (2012) also pointed out the individual’s psychological feelings can be affected by proactive coping strategies. The significance of proactive coping as an indicator of life satisfaction among undergraduate freshmen was shown in the study by Dwivedi and Rastogi (2017). All the above are in line with the significant path of the present study that wisdom→ proactive–reflective coping →adaptation (social and psychological adaptation) and life satisfaction. For the Chinese visiting scholars, adopting proactive–reflective coping strategies can help them enhance the planning and reflective ability to handle future challenges in the workplace and social context in Canada while improving their psychological wellbeing.

However, the effect of passive coping was only significantly when mediating between 3DWS, adaptation, and life satisfaction, and there was no significant mediating effect of active coping between wisdom (3DWS, ASTI), adaptation, and life satisfaction. This is possible because the investigation was implemented during the COVID-19 and the stressful events they encountered were in close relation to the pandemic. Personal wisdom is considered a quality or aspect of the person, whereas the actual use of coping strategies is a process that is not only influenced by individual qualities, but also by the context (Aldwin, 2007). Both active coping and passive coping are responses to stressful events that have already occurred (Greenglass, 2002), and the selected coping strategies the participants used will depend on how it is being applied in the face of the stressor. In the context of COVID-19, Chinese visiting scholars were all facing unexpected challenging events and were unable to respond to related stressors directly. The more adaptable individuals might be the ones who prepared themselves in advance. Thus, the mediating effect of active coping and passive coping were insignificant or only partially significant between wisdom, adaptation, and life satisfaction.

There are some limitations in the presented work that need to be addressed in the future. First, the concurrent influences on adaptation and life satisfaction were examined in a single testing occasion, which makes it difficult to determine the developmental pathways or draw causal conclusions from the relationship between wisdom, coping styles, adaptation, and life satisfaction in the interpretation of results. In the future, more longitudinal, experimental, or intervention studies could be employed to provide more information about within- and between-person differences in the relationship between wisdom, coping, and adaptation. Second, the generalizability of the findings is limited due to the relative homogeneity within this sample. All of our participants were Chinese visiting scholars in Canada. Future investigations should be broadened to other acculturation groups living in other countries and explore whether the relationships among wisdom, coping styles, adaptation, and life satisfaction found in the present study can be replicated. Third, on the basis of quantitative research, biographical interviews can help to explore in-depth personal wisdom and visiting scholars’ experience and elucidates within-person change.

Despite the limitations, the findings in this study still make a significant contribution to practice and research. First, the insight into the psychological mechanism underlying the association between wisdom and adaptation was substantially extended. In addition, one of the most intriguing findings is the significant path from wisdom to social, psychological adaptation, and life satisfaction, through proactive–reflective coping, which suggests that proactive–reflective coping may be an important mediating variable between wisdom, adaptation, and life satisfaction. It echoes a previous finding where proactive coping not only relates to depression negatively (Mohammad and Zahra, 2012) but also predicts positive results and promotes wellbeing (Greenglass and Fiksenbaum, 2009). Further research should examine wisdom and coping styles in more detail with sub-samples in order to determine the relations between wisdom and many other types of coping. Additionally, unraveling the relationships between wisdom, coping styles, and adaptation may enrich the theories of wisdom and adaptation based on different cultural background. The 3DWS and the ASTI were independent predictors of these coping and outcome measure, which reinforce that the notion that these scales are tapping different but equally important aspects of wisdom. Second, the findings in the study indicate that proactive–reflective coping strategies could help visiting scholars adapt more easily to life in Canada. Therefore, intervention programs focusing on improving or training their proactive coping skills might mitigate negative influences of psychological distress and enhance the quality of life in adaptation. In turn, it might help develop their wisdom when they learn to cope with their life challenges during the adaptation process. Lastly, this is a new study about the visiting scholars, which provides a new perspective for the related research of this group of people. It is better to start with a single group, but are there experiences typical for other groups, that can be comparable experiences for future direction of research.

In conclusion, the study seeks to contribute to investigating the relationship between wisdom and adaptation. It further suggests that wisdom, as a useful construct in the study of adaptation and acculturation, maybe a major contributor to positive outcome. Also, it reveals that individuals who report a higher level of wisdom may engage more in proactive–reflective coping strategies and are more satisfied with their lives. It is hoped that the present empirical study will inspire further discussions and debates on the wise-coping approach to adaptation and serve to stimulate further study to understand wisdom and its relationship to the adaption process and outcome. Besides, it is estimated that wisdom and adaptation might reciprocally interact with each other. Sophisticated study designs, such as cross-lagged panel studies, would help explore how mutual influence unfolds over time. All the answers to these questions will have far-reaching implications for practice, research, and policy-making in the future.

## Data Availability Statement

The raw data supporting the conclusions of this article will be made available by the authors, without undue reservation.

## Ethics Statement

The studies involving human participants were reviewed and approved by the Hubei University of Medicine Ethics Committee. The patients/participants provided their written informed consent to participate in this study.

## Author Contributions

DB, LZ, MF, and ZF contributed to conception and design of the study. DB, YC, and LZ organized the database and performed the statistical analysis. DB wrote the first draft of the manuscript. MF provided resources, supervision for the study, and reviewed the manuscript. MF, DB, and ZF contributed to manuscript revision. All authors contributed to the article and approved the submitted version.

## Funding

This research was funded by the Humanities and Social Science Research Projects of Hubei Province (grant no: 21Y162).

## Conflict of Interest

The authors declare that the research was conducted in the absence of any commercial or financial relationships that could be construed as a potential conflict of interest.

## Publisher’s Note

All claims expressed in this article are solely those of the authors and do not necessarily represent those of their affiliated organizations, or those of the publisher, the editors and the reviewers. Any product that may be evaluated in this article, or claim that may be made by its manufacturer, is not guaranteed or endorsed by the publisher.

## Footnotes

^1^https://opendoorsdata.org/
